# Nudges in a post-truth world

**DOI:** 10.1136/medethics-2017-104153

**Published:** 2017-05-19

**Authors:** Neil Levy

**Affiliations:** 1 Department of Philosophy, Macquarie University, Sydney, New South Wales, Australia; 2 Uehiro Centre for Practical Ethics, University of Oxford, Oxford, UK

**Keywords:** Autonomy, Behaviour Modification

## Abstract

Nudges—policy proposals informed by work in behavioural economics and psychology that are designed to lead to better decision-making or better behaviour—are controversial. Critics allege that they bypass our deliberative capacities, thereby undermining autonomy and responsible agency. In this paper, I identify a kind of nudge I call a nudge to reason, which make us more responsive to genuine evidence. I argue that at least some nudges to reason do not bypass our deliberative capacities. Instead, use of these nudges should be seen as appeals to mechanisms partially constitutive of these capacities, and therefore as benign (so far as autonomy and responsible agency are concerned). I sketch some concrete proposals for nudges to reason which are especially important given the apparent widespread resistance to evidence seen in recent political events.

A number of different sources have proclaimed that the world has entered a ‘post-truth’ era. Indeed, ‘post-truth’ was named the word of the year by Oxford dictionaries.^1^ Plausibly, we are in a post-truth era not because people no longer care about the truth, but because the beliefs of many are not responsive to the best evidence. Perhaps this phenomenon arises from changes in people's psychology (eg, greater anxiety might make people more susceptible to affective influences and less susceptible to argument). Alternatively, it might arise from changes in the external environment (eg, perhaps the phenomenon is explained by the decline of the traditional press and the rise in fake news sites). No matter the explanation, it poses one of the most important problems facing us today. If the promise of democracy is to be sustained, people's decisions, and therefore their beliefs, must be guided by evidence. Increasingly, across a range of issues, people seem to make up their minds in ways that are at variance with the evidence apparently available to them.

If it is indeed true that we have witnessed a significant change in people's responsiveness to evidence, the phenomenon itself is far from new: rather, the change consists in the generalisation of a long-established phenomenon. Take, for one important example, the protection of children and adults from infectious diseases for which there are effective vaccinations. Because some people cannot be vaccinated for medical reasons, and some are too young for vaccination, widespread vaccination is a social and an individual good: widespread vaccination leads to ‘herd immunity’, which is a social good accruing to the vaccinated and the non-vaccinated alike. But an increasing number of people today refuse vaccination. The scare caused by Andrew Wakefield's infamous and fraudulent linking of vaccination to autism has never receded, and many parents remain convinced that vaccinations represent a risk to their children and resist the evidence that their fears are misplaced.

The phenomenon of motivated resistance has attracted a great deal of attention from psychologists. They have studied the correlates and causes of such resistance, and they have examined how false beliefs might be corrected. Some of their findings are depressing (indicating that people may actually become *more* entrenched in false beliefs when presented with good evidence against them), but some offer hope. The aim of this paper is to survey some of this work, preparatory to assessing the ethical permissibility of using suggestions for addressing false beliefs that arise from it. The proposals that arise may be seen as belonging to the broader class of ways of affecting behaviour that have come to be called *nudges*. Nudges are controversial for several reasons, but the central objection to them, and the objection on which I will focus here, is that they are unacceptably paternalistic and therefore threaten the autonomy of agents. Autonomy is very plausibly a great good, so even if nudges conduce to the well-being of the nudged or to social goods, they may be impermissible. In this paper, I identify a class of nudges that I call *nudges to reason*. I argue that these nudges do not threaten our autonomy. Interventions into decision-making and belief formation threaten our autonomy when they bypass our capacities for deliberation. Nudges to reason do not bypass our capacities for deliberation. Rather, they address themselves to capacities that are partially *constitutive* of our reasoning. There are therefore strong reasons to think that nudges to reason are permissible.

In the first section of this paper, I will set out some of the evidence from psychology for motivated resistance to evidence, and for ways in which this resistance can be overcome. The second section turns to suggestions, also arising from psychology, for increasing genuine responsiveness to evidence. Since these suggestions seem to constitute nudges, I turn to objections to nudges, and specifically the objection that they undermine our autonomy by bypassing our capacities for deliberation. I argue that the nudges that have been shown to be effective in increasing our responsiveness to reason are addressed to, rather than bypassing, our deliberative capacities.

## The limits of argument

There is a great deal of evidence that giving people strong arguments to change their minds often fail to work when people are motivated to reject the evidence. In fact, those who are motivated to reject the claims may become *more* entrenched in their views than previously. This is known as the *backfire effect*. For a famous illustration, consider Nyhan and Reifler.^2^ They gave their participants mock news articles, which included a genuine quote from President Bush implying that Saddam Hussein had possessed weapons of mass destruction (WMD) at the time of the 2003 US invasion. In one condition, the participants received versions of the story which contained an authoritative correction (from the Duelfer Report), indicating that Saddam had no WMD programme and no stores of WMDs at the time of the invasion. Participants were then asked to indicate their level of agreement with the claim that Saddam had stockpiles of WMDs and an active WMD programme just prior to the invasion. Participants were also asked to report their political orientation, on a 7-point scale, ranging from ‘strongly liberal’ to ‘strongly conservative’. For those participants who placed themselves on the right of the scale, the correction backfired: they expressed stronger agreement with the claim that Saddam had an active WMD programme and stockpiles of WMDs than conservatives who did not receive a correction.

The backfire effect has been documented many times. Even when corrections are effective in reducing (reported) belief strength, they may have a backfire effect on behaviour: Nyhan *et al* 3 found that correcting the myth that vaccines cause autism was effective at the level of belief, but actually *decreased* the intention to have one's children vaccinated among parents who were initially least favourable to vaccines. Nyhan and Reifler^4^ documented the same phenomenon with regard to influenza vaccines.

What explains the backfire effect? Peter and Koch^5^ argue that it arises as a consequence of misremembering. Memory distortions almost certainly play a role, as they suggest. Memory traces may decay at different rates; source claims (‘vaccines cause autism’) and discounting contextual information (‘according to a retracted paper’) may therefore come to be dissociated in retrieval. That is, people may recall the first without recalling the second. If the claim is especially striking, perhaps because it carries information about risks to oneself or to loved ones, it may decay more slowly or be more salient and therefore be more easily recalled (this might help explain the persistence of conspiracy theories and urban legends in the face of being debunked: claims like ‘the CIA engineered the collapse of the Twin Towers’ or that ‘there are alligators living in the sewers of New York City’ are extremely striking and may have an advantage in contests for access over pallid, boring, discounting sources).

There is evidence supporting the claim that the backfire effect is at least in part the result of memory distortions. Skurnik *et al* 6 demonstrated that when participants were given claims about health and nutrition, with each claim labelled either ‘true’ or ‘false’, participants were more likely to misremember false claims as true than vice versa after a delay. The fact that the backfire effect is more potent after a delay of at least several days^7^ also suggests that misremembering plays an important role in its production. But misremembering is far from the whole story. Processing fluency—the subjective ease of recalling and manipulating the information—also plays an important part. When claims are processed fluently, they are more likely to be believed. Disfluency, on the other hand, is a metacognitive signal that something is not right, and triggers analytic processing.^8^ ^9^ A variety of different factors affect fluency.^10^ Here I mention just two, of particular relevance for failures of correction. Claims that are intuitively plausible are processed more fluently than those that are not (which often puts scientific findings at a disadvantage, because they are often quite unintuitive^11^). Claims that are repeated are processed more fluently; that entails that mere repetition of a claim—in the service of debunking it, for instance—may increase its plausibility.

Fluency effects may support or explain misremembering. Perhaps some claims are more easily remembered because they are processed more fluently. But fluency may also explain failures of correction in the absence of misremembering. Someone may recall the discounting cue as well as the claim, but devalue the former because the claim itself is processed so fluently. An alternative mechanism for the backfire effect is the motivated processing of information. There is plentiful evidence that people are in general very much better at detecting—and also imagining—problems with arguments for claims that they are motivated to reject than for claims they are motivated to accept. The classic demonstration of this asymmetry involved giving participants two sets of evidence, one supporting the claim that capital punishment is an effective deterrent and one supporting the claim that it is not; the sets were constructed so that the cases were equally strong.^12^ People who have strong views about capital punishment might be expected not to shift in the face of this equivocal evidence. In fact, they did shift, becoming *more* convinced of their antecedent view. It is the asymmetrical scrutiny of evidence that seems to underlie this result. After scrutiny of the evidence, participants take themselves to be in possession of some extra, genuinely strong, evidence supportive of their antecedent view, and no persuasive evidence at all against it. They therefore become more convinced. This may be seen as a version of the backfire effect, because it involves people presented with mixed evidence, which might be expected to moderate their views, becoming more extreme instead.

The evidence just surveyed suggests that we are much less responsive to evidence than we might have hoped. Indeed, we are often *perversely* responsive to evidence, becoming firmer in beliefs when presented with strong evidence against them. Our lack of responsiveness, or perverse responsiveness, may play an important role in explaining recent political events, as well as such social problems as those arising from vaccine refusals. These mechanisms may explain why we find ourselves in the current post-truth age, if that is indeed an accurate characterisation of contemporary times. But the news is not all bad. Psychologists have also identified a number of ways in which our responsiveness to reason might be improved. In the next section, I will outline one of their suggestions, prior to turning to an assessment of its ethical permissibility.

## Avoiding backfire

While there are a number of strategies for improving responsiveness to evidence, in this section I will focus on those that take advantage of our growing knowledge of how we respond to testimony. There is a rapidly growing literature (largely, although not exclusively, developmental) devoted to understanding the conditions under which we take other agents' word for claims.^13^ Children and adults must learn from others: there is a great deal that we *cannot* check for ourselves, and a great deal more that it would be too time-consuming or otherwise costly to check. In the contemporary world, we rely on medical specialists to diagnose our ills, technology specialists to fix our computers, accountants to manage funds for our retirement and meteorologists to advise us when to hold a picnic. But this reliance on specialists is by no means confined to modernity: the division of labour, including cognitive labour, is a feature of traditional societies too.^14^ Canoe making, for instance, is a specialised skill, and not everyone has the time to acquire it. Moreover, skill acquisition is itself dependent on the acceptance of testimony: children often cannot discover essential techniques for survival themselves, and must be taught them. Sometimes, there is a large temporal gap between initiation of training and sufficient acquisition of the skill to be able to judge for oneself that the techniques being taught are indeed appropriate for the ends sought. For all these reasons, we are often forced to learn from others in the absence of a capacity directly to gauge how reliable they are. We are therefore forced to use cues to reliability; cues which reliably enough correlate with being a good source of testimony.

Many of these cues are quite commonsensical. Unsurprisingly, children are sensitive to evidence that the informant is reliable on other matters. Since there is often a correlation between being accurate on one subject matter and another, using cues for accuracy is a good heuristic. Use of this heuristic becomes more sensitive with age: younger children are reluctant to accept any testimony from someone who has been wrong in the past, whereas older children calibrate their trust in ways that are more sensitive to statistical accuracy.^15^ Children are also sensitive to evidence of the benevolence of informants, and reluctant to accept testimony from those with a track record of malevolence. This, too, is unsurprising. If sensitivity to the past accuracy of informants provides protection against being deceived by fools, sensitivity to the benevolence of informants provides protection against being duped by knaves who might exploit us for their own ends.

Sensitivity to cues of benevolence helps to explain why some corrections are successful, I suggest. Several researchers have found that the source of a correction makes a significant difference to whether it is effective. For instance, Nyhan and Reifler^16^ found that both the perceived ideological leanings of the media outlets doing the reporting (eg, FOX vs MSNBC) and of the debunking claim reported (a liberal think tank vs a non-partisan vs a conservative think tank) made a significant difference to the extent to which corrections of myths about Obama's policies were effective for conservatives. When a rebuttal was reported by a liberal news channel and sourced to a liberal think tank, conservatives were subjected to a backfire effect (inasmuch as their attitudes towards Obama became more negative), but when the news channel and the source were conservative, the correction was effective. A likely explanation, I suggest, is that conservative sources pass the tests for general reliability and for benevolence deployed by conservative information consumers; their conservative credential entail that they share both normative and factual orientations with their audience. But corrections need not come from sources that share one's ideological orientation to be effective: Berinsky^17^ found that corrections made by sources who can be expected to find the claim they affirm contrary to their own ideological interests are effective, both for those who share the source's ideology and those who reject it. Thus, corrections to myths about Obamacare that stem from Republican sources are effective for liberals and conservatives alike; the fact that the claim is contrary to the source's interests is taken to be evidence in its favour.

Thus, one way to raise the likelihood that agents respond as they rationally ought to corrections is to seek out authoritative corrections from sources that either share the ideological orientation of their audience, or can be expected to find the claim affirmed unpalatable (often potential sources will have both properties: thus, an authoritative correction of a myth about a contentious policy that comes from an opponent of the policy can be expected to be especially effective). Such corrections will be most powerful if they are reported as fact by media that can also be expected to find them unpalatable. There are other ways of making corrections more effective (eg, ways that are sensitive to the framing of claims, or which provide alternative causal explanations for observed facts^16^), but this example will serve for our purposes.

Whatever one's views on the recent US election or the Brexit referendum in the UK, there can be no doubt that resistance to evidence is responsible for large-scale social problems. From the loss of herd immunity to the decline in the quality of science education in many countries to the failure to address climate change, lack of responsiveness to strong evidence underlies many ills. The evidence just reviewed suggests that there may be ways to address these problems, increasing the degree to which people form beliefs in ways that are sensitive to the actual drift of the available evidence. Since the social problems are very significant, such proposals obviously have a great deal in their favour. But that is not sufficient to entail that they are all things permissible. We might see the recommendations just surveyed as nudges, and nudges are controversial. In the next section, I outline some of the controversy, with a view to assessing the overall permissibility of these nudges.

## Nudges and autonomy

The previous section surveyed some of the evidence that backfire effects can be avoided: even when people are motivated to accept a false claim, they can be brought to respond appropriately (ie, altering their credences in the direction of the overall drift of the evidence, if not always coming to hold the view that is best supported by the evidence), if the evidence is presented in certain ways. Some social scientists have explicitly urged that these ways of presenting evidence should be used to counteract public ignorance and misconceptions.^16^

Given the apparent influence of fake news, urban legends and deliberate lies on recent elections and referendums, there is significant potential for these interventions to lead to better decision-making on the part of the public. Very plausibly, their adoption would be in the interests of those they affect. When it comes to assessing the permissibility of such interventions, evidence that interventions would lead to an improvement in overall well-being is obviously important. But such improvements are not the only thing that matters; even consequentialists will be concerned with the broader effects of such interventions (eg, on capacities for decision-making more generally), while deontologists and virtue ethicists will have other concerns.

Interventions like those suggested seem to belong to the class of what has come to be called nudges.^18^ Nudges are proposals for policy aimed at improving well-being and enhancing decision-making that are inspired by work in behavioural economics and social and cognitive psychology. Thaler and Sunstein argue that (many) nudges are permissible: they differ from impermissible paternalistic interventions in that they leave agents free to choose. Changing the default options on insurance policies, for example, leave consumers free to choose any of the options; the intervention just makes it more likely that they will choose the option that is the new default (perhaps due to status quo bias). Similarly, ensuring that healthy food options, and not junk food, are at eye level leaves consumers free to choose the junk if they wish; again, the intervention simply makes it more likely that people will choose the now salient healthy option. Because these interventions aim at enhancing the well-being of those they affect but leave them as free to choose as they would have been without them, Thaler and Sunstein call a programme of using them libertarian paternalism.

While almost everyone accepts that libertarian paternalism is preferable to more coercive varieties, many people doubt the libertarian credentials of at least some of the nudges that Thaler and Sunstein advocate. As Saghai^19^ notes, there are two senses in which nudges may be said to preserve freedom of choice, which we may call a basic sense and a substantive sense. It is clear that nudges preserve freedom of choice in the basic sense: they do not foreclose options. Rather, they ‘nudge’ agents from some options towards others. But critics worry that they do not preserve freedom in a more substantive sense. These interventions bypass agents' capacities for deliberation. They do not address arguments to us; rather, they take advantage of non-rational features of our nature (such as our reliance on the status quo bias or on the salience of options) to produce their effects. To the extent to which they bypass our capacities for responding to reasons, they should be regarded with suspicion, these critics suggest. They are *pro tanto* (if not always all things considered) wrong to this extent.^20^ ^21^

Why are interventions *pro tanto* wrong to the extent to which they bypass our capacities for deliberation? There are several, interlinked, reasons to regard such bypassing with suspicion. Prima facie at least, we owe one another a certain distinctive kind of respect—the respect due to persons alone—in virtue of our being rational agents, and we manifest this respect by addressing one another *as* rational agents. To the extent to which we circumvent powers of rational assessment, we fall short of manifesting this kind of respect. There is a close connection between this kind of respect and responsibility. On many accounts, moral responsibility is essentially linked to reasons-responsiveness: agents are morally responsible for their actions only when these actions are caused by reasons-responsive mechanisms.^22^ To circumvent our rational capacities is therefore to fail to treat one another as responsible agents, and it is perhaps for this reason that it fails to treat us with the respect rational agents deserve.^23^ Conversely, addressing one another as rational agents may actually promote moral responsibility: it enables the development of capacities for assessing and responding to reasons as reasons, and thereby brings it about that the sphere in which we are capable of taking responsibility expands. Any intervention that threatens our substantive freedom is an intervention that undermines our responsibility, and thereby fails to treat us as autonomous agents deserving of respect. While there are other reasons to worry about nudges, it is this kind of worry that has occupied centre stage in the debates over their permissibility, and it is this kind of worry on which I will focus here.

Nudges that aim to increase our responsiveness to evidence are distinctive in some ways. I will call this class of nudges *nudges to reason*. Unlike some other nudges, nudges to reason do not affect behaviour directly or in ways mediated by the non-rational elements of mind (such as affect, on some ways of understanding emotions). Rather, they affect behaviour in ways that are mediated by beliefs. They change behaviour by changing minds (in the same way in which arguments—the presentation of evidence for a proposition—change behaviour by changing minds). But not just any nudge that changes minds (and thereby behaviour) is a nudge to reason. A nudge qualifies as a nudge to reason when it changes minds by making them more responsive to genuine evidence.

Despite the fact that nudges to reason aim at changing minds in line with the rational significance of genuine evidence, there may nevertheless be something objectionable about *how* they change minds. While critics may concede that insofar as nudges to reason make us more sensitive to the genuine force of evidence, they may argue that the interventions are designed to take advantage of our cognitive natures in ways that bypass our deliberative faculties. Evidence has the same rational weight whatever its source; hence ensuring that the source, or the news channel reporting them, is chosen to avoid the backfire effect involves interlocking with non-rational elements of mind, it may be claimed.^i^ Hence, nudges to reason may be seen to raise the same worries, perhaps in an attenuated form, as other nudges. By bypassing our deliberative capacities, they may threaten the substantive freedom of our choices even if they succeed in making us more responsive to the evidence.

One possible reply to this worry is to claim that nudges to (or away from) reason are inevitable.^24^ ^25^ Claims inevitably have sources: they are reported by media that are liberal, conservative or non-partisan (all of which have predictable effects on the weight given to them);^16^ and the ideological orientation of the media is typically known by their audience. In those cases in which source ideology is not be known, the audience will likely infer ideology from cues (linguistic, sartorial, accent) and from the content of the report. We are unlikely to be able to avoid triggering the mechanisms that filter testimony to assess its plausibility. It may be the case, in fact, that these mechanisms are a proper part of the reception and assessment of testimony, and cannot be avoided. If we know that testimony source will inevitably have effects on how arguments are processed, then the objection that our use of this knowledge bypasses agents' capacities for deliberation is considerably weakened. Perhaps nudges to reason bypass these capacities, but we cannot avoid this kind of bypassing and the fact that they take advantage of these mechanisms should not count against them.

While the unavoidability argument has a great deal of force, I think there is a stronger reply available. We should deny that nudges to reason bypass the deliberative capacities of agents at all. The proposed interventions are designed to be processed by filters that are partially *constitutive* of reasoning in normal functioning agents, not an obstacle to reasoning, or even merely a brute support of reasoning. Thus, designing arguments to appeal to them is not bypassing reasoning, but appealing directly to it. Addressing the mechanisms at issue is appealing to reason, in a way that is analogous to the way in which giving inductive evidence for a claim is appealing to reason.^ii^

The fact that the mechanisms appealed to by nudges to reason are partially constitutive of reasoning is easily overlooked, because we tend to identify ‘reasoning’ with ‘conscious reasoning’. We therefore implicitly use a test along the following lines to assess whether an element in the causal pathway leading to a judgment involves reasoning: would the person endorse its influence were she aware of it? This test often yields the right result (eg, distinguishing between cases of indoctrination and those of mere influence^27^). But the test often goes astray: much of our reasoning, including our conscious reasoning, involves processes that are opaque to introspection, and our naïve theories about how we reason are often false. We may therefore come to regard constitutive elements of our reasoning as alien to it.

Consider the influence of affect on cognition. Many people regard emotion as at best irrelevant to, and at worse as distorting, good reasoning. But affective responses are actually, although only partially, constitutive of good reasoning.^28^ They orient us towards some responses and away from others, thereby *enabling* deliberation: we would face an intractable problem of combinatorial explosion if implicit processes did not play this orienting and winnowing role for us. Damasio^29^ has presented evidence how emotional deficits lead to difficulties in decision-making, ranging from paralysis in the face of some easy tasks to bad decisions on others. Of course, emotions do not *always* lead to better decisions. The naïve view is quite right that sometimes strong emotions may cause people to take unjustifiable risks, overlook important options and so on. But the fact that affect sometimes causes bad decisions is not grounds for holding that it is not partially constitutive of reason: that would entail holding it to a standard that *nothing* meets. Conscious reasoning, too, may mislead: sometimes we perform better when we rely on intuition or heuristics than on explicit deliberation. When we have expertise in a domain, our quick, intuitive, judgment is often better than slower reflection.^30^ Since it is unreasonable to identify reasoning with processes that always lead to better outcomes, we should instead hold processes to a lower standard to assess whether they are constitutive of reasoning.

A process is a proper part of reasoning, I suggest, when it regularly and reliably supports better deliberation (either in a domain-general or a domain-specific manner). While we may investigate whether a process passes this test by gathering systematic data, we are often in a position to be confident that processes will pass it without the need for investigation. If we have good reason to believe that a particular mechanism is part of our evolved psychology, and that it is designed to contribute to information processing, then we should expect it to be partially constitutive of reasoning. Since reasoning is adaptive, for obvious reasons, mechanisms that cause us to reason badly will tend to be weeded out by natural selection. Admittedly, there are significant difficulties with this evolutionary justification for confidence that a mechanism is partially conducive of deliberation. Mechanisms may be deliberation-conducive only in the domains for which they are designed, and it is often tricky to delineate the domain. Nevertheless, matters are often clear enough that appeal to this evolutionary justification is sufficient to justify confidence in the claim that a particular mechanism is constitutive of reasoning.^iii^

Appeals to the mechanisms that weigh testimony by reference to their source are very plausibly appeals to mechanisms that are partially constitutive of rationality, because we likely have such mechanisms in virtue of the role they played in enabling better decision-making. As we saw above, these mechanisms are sensitive to the previous track record of the source. That is, very obviously, sensitivity to a property that is truth-conducive. We *should* put less weight on the testimony of those who are frequently wrong than those who have better records. Similarly, sensitivity to the ideological orientation of the source is also truth-conducive. We should be wary of the claims of people who lack benevolence towards us, because they may be motivated to exploit us. We also should put more stock in testimony from agents who have an incentive to reject the claim they affirm. Thresholds for being convinced of a claim are sensitive to the stakes for the person: it takes weaker evidence to convince people of claims they are motivated to accept than claims they are motivated to reject.^12^ ^32^ When someone testifies that a claim is true and we have good reason to think that they are motivated to reject the claim, we should think that the evidence in favour of the claim is especially strong. Sensitivity to these properties is sensitivity to considerations that are relevant to the credence we should place on testimony. Appealing to them is appealing to capacities that have as their proper function the assessment of reasons for belief—a function that is obviously partially constitutive of reasoning—in their role as reasoning mechanisms.

Nudges to reason are therefore appeals to our deliberative capacities. But if appeals to these mechanisms are appeals to our deliberative capacities, we should not think that in so appealing we fail to treat one another as responsible or autonomous agents. Nor should we think that appeals like these limit our substantive freedom; arguably, our freedom *consists in* the capacity to respond and react to reasons.^22^ Appeals to these capacities therefore enable responsible decision-making on the part of the agents whose capacities they are. As far as this set of interlinked considerations (autonomy; responsibility; dignity; freedom) are concerned, there is no reason to worry about nudges to reason. We should think that such nudges are permissible (perhaps we even have an *obligation*, stemming from the respect we owe to rational agents, to frame our arguments such that are maximally truth-conducive, and therefore to make such appeals).

That is not to say that all nudges that make us more sensitive to genuine evidence to reason are appeals to our deliberative capacities. There may be some that bypass these capacities. Some nudges might make us more sensitive to reason but fail to work through mechanisms partially constitutive of our rationality. Consider, for example, a mechanism that generates a preponderance of false positives, because in the environment of evolutionary adaptiveness false negatives were very costly and false positives very cheap. Such a mechanism is an adaptation, but plausibly it is not an adaptation *for reasoning*. It does not, regularly and reliably, allow us to track the truth. A nudge that appealed to this mechanism might, in particular instances, make us more sensitive to the actual reasons prevailing, but because the mechanism is not a proper part of our deliberative capacities, it could not be defended against worries concerning autonomy and responsibility on the grounds adduced here.^iv^

## Conclusion

In this paper, I have identified a class of nudges, I call nudges to reason. Nudges to reason are distinguished from other nudges by the fact that they attempt to change behaviour by changing minds, and they change minds by making us more responsive to the genuine force of reasons. Perhaps there are nudges that make us more sensitive to genuine evidence that work by bypassing our deliberative capacities, but at least some such nudges appeal to capacities that are partially constitutive of these capacities. There is therefore no more reason to worry that such nudges undermine our autonomy or responsible agency than that arguments generally threaten these things.

## Footnotes

i Of course, expert testimony often carries great weight. But we are interested in the difference in persuasive power when a claim is made by a conservative versus a liberal, say, and not an expert versus a non-expert.

ii Grüne-Yanoff and Hertwig^26^ reserve the term ‘nudge’ for interventions that treat agents as instinctive automatic systems. They might therefore prefer their term ‘boost’ for interventions that make agents more responsive to evidence. I do not adopt this terminology, in part because I reject their further claim that boosts stem from a different research tradition to the paradigm nudges, or that the latter rarely make agents more responsive to evidence.

iii Grüne-Yanoff^31^ also draws attention to the nature of the mechanisms underlying nudges in assessing whether a nudge will be effective in bringing about its goals in a particular environment; as he notes, the same effect can be brought about by quite different mechanisms. While my defence of nudges to reasons requires that such nudges are implemented by certain mechanisms and not others, it does not require that the nudge be implemented by any *specific* mechanism. I require, rather, that it be implemented in a way that entails that it is a constituent of reasoning. The considerations mentioned in this paragraph provide a defeasible reason for believing that such nudges are implemented in the right kind of way. I thank a reviewer for drawing this paper to my attention.

iv It may be that the defence of nudges to reason offered here may be extended to other nudges. Thaler and Sunstein suggest that we may prefer default options because we are disposed to take the identification of a default as endorsement; if the disposition is truth conducive, the defence might succeed for this nudge.
